# Complete Mitochondrial Genomes of *Nannostomus* Pencilfish: Genome Characterization and Phylogenetic Analysis

**DOI:** 10.3390/ani14111598

**Published:** 2024-05-29

**Authors:** Wei Xu, Jingzhe Tai, Ke He, Tangjun Xu, Gaoji Zhang, Boyu Xu, Hongyi Liu

**Affiliations:** 1 The Co-Innovation Center for Sustainable Forestry in Southern China, College of Life Sciences, Nanjing Forestry University, Nanjing 210037, China; xuwei2001@njfu.edu.cn (W.X.); xiaotai@njfu.edu.cn (J.T.); xutangjun@njfu.edu.cn (T.X.); zhanggaoji@njfu.edu.cn (G.Z.); xuboyu@njfu.edu.cn (B.X.); 2 College of Animal Science and Technology, Zhejiang Agriculture and Forestry University, Hangzhou 311300, China; heke@zafu.edu.cn

**Keywords:** aquarium fish, mitochondrial genomes, *Nannostomus*, pencilfish, phylogenetic analysis

## Abstract

**Simple Summary:**

To complement the genetic information of the pencilfish, a popular aquarium ornamental fish, we sequenced the mitochondrial genomes of four common pencilfish species. Their genome structure, nucleotide composition, codon usage, and phylogeny were comparatively analyzed. The results indicate that the four mitogenomes exhibited a typical circular structure. The gene order of the four *Nannostomus* pencilfish was similar to that of other fish. Our phylogenetic analyses support the current classification of the family Lebiasinidae. This study provides new data for the breeding and study of pencilfish.

**Abstract:**

Although the pencilfish is a globally popular economic fish in the aquarium market, its taxonomic classification could be further refined. In order to understand the taxonomy of species of the genus *Nannostomus* (Characiformes, Lebiasinidae) and their phylogenetic position within the order Characiformes, in this study, we characterized mitochondrial genomes (mitogenomes) from four *Nannostomus* species for the first time. The four mitogenomes exhibited the typical circular structure, with overall sizes varying from 16,661 bp to 16,690 bp. They contained 13 protein-coding genes (PCGs), 2 ribosomal RNA genes (rRNAs), 22 transfer RNA genes (tRNAs), and 1 control region (CR). Nucleotide composition analysis suggested that the mitochondrial sequences were biased toward A and T. Bayesian inference and maximum likelihood analyses based on PCGs support the family Lebiasinidae classification, described using four *Nannostomus* species, clustering together with *Lebiasina multimaculata* from the same family. The results of this study support the current taxonomic classification of the family Lebiasinidae. Phylogenetic analysis also suggested that gene rearrangement would not significantly impact the phylogenetic relationships within the order Characiformes. These results might provide new data regarding the phylogeny and classification of the order Characiformes, thus providing a theoretical basis for the economic development of aquarium fish markets.

## 1. Introduction

The family Lebiasinidae, which belongs to the order Characiformes, consists of over 70 valid species of small freshwater fish that are widely distributed across South and Central America, spanning from Costa Rica to Argentina [1]. Lebiasinidae is divided into two subfamilies, Lebiasininae (which includes the genera *Derhamia*, *Lebiasina*, and *Piabucina*) and Pyrrhulininae (which includes the genera *Copeina*, *Copella*, *Nannostomus*, and *Pyrrhulina*) [2]. The latter represents the most diverse clade, and the genus *Nannostomus* is the most species-rich genus in the subfamily [2]. Most species in *Nannostomus* are slender and pencil-shaped, with lengths ranging from 1.5 cm to 7 cm, and are highly popular in the aquarium market under the popular name “pencilfish” [1,3,4]. The global aquarium trade has up to 5300 freshwater fish species available for sale each year, with about 1 billion individuals [5]; of these, species in the order Characiformes account for a certain share [1,4]. Despite its economic importance, there have been incomprehensive reports about its basic biological data, including genetic information [3,6]. Given the wide variety of species, diverse body colors, and limited gene sequence databases, understanding the taxonomy of species in the genus *Nannostomus* poses a significant challenge [2]; the phylogenetic position of family Lebiasinidae within the order Characiformes could be further refined [7]. The phylogenetic position of Lebiasinidae within the order Characiformes has been a topic of frequent discussion [8,9].

In the realm of animals, the mitochondrial genome (mitogenome) is a small, circular genome, ranging in size from 15 to 18 kb [10,11]. It generally contains 13 protein-coding genes (PCGs), 22 transport RNA (tRNA) genes, 2 ribosomal RNA (rRNA) genes, and 1 control region (CR) [12,13]. The 13 PCGs are *NADH dehydrogenase subunit 1* (*ND1*), *NADH dehydrogenase subunit 2* (*ND2*), *Cytochrome c oxidase subunit I* (*COX1*), *Cytochrome c oxidase subunit II* (*COX2*), *ATP synthase F0 subunit 8* (*ATP8*), *ATP synthase F0 subunit 6* (*ATP6*), *Cytochrome c oxidase subunit III* (*COX3*), *NADH dehydrogenase subunit 3* (*ND3*), *NADH dehydrogenase subunit 4L* (*ND4L*), *NADH dehydrogenase subunit 4* (*ND4*), *NADH dehydrogenase subunit 5* (*ND5*), *NADH dehydrogenase subunit 6* (*ND6*), and *Cytochrome b* (*Cytb*) (arranged in the common order in the mitogenome of the order Characiformes) [12,13]. The utilization of mitogenomes in molecular identification and phylogenetic analysis is prevalent due to their rapid evolution rate, simple structure, low molecular weight, and maternal inheritance [14,15]. Certain mitochondrial gene fragments, namely *16S rRNA*, *COX1*, and *Cytb*, have been extensively employed in phylogenetic analyses [16,17]. However, the utilization of partial mitochondrial sequences is constrained by their limited capacity to provide comprehensive information [11]. On the other hand, the complete mitogenome offers a higher level of resolution and sensitivity, making it more suitable for the examination of phylogenetic relationships and species classification [18,19].

With the application of next-generation sequencing technology, there has been a growing number of mitogenomes sequenced in recent years [20]. However, only a limited number of complete mitogenomes are available in the family Lebiasinidae [3,6], and no complete mitogenome is available in the genus *Nannostomus*. In this study, we present the complete mitogenomes of four *Nannostomus* species, namely *Nannostomus beckfordi*, *Nannostomus marilynae*, *Nannostomus marginatus*, and *Nannostomus unifasciatus*. Specifically, the mitochondrial characteristics of these four species, including their gene order, genome size, nucleotide composition, codon usage, and tRNA secondary structure, are comparatively analyzed with other species within the order Characiformes. This study provides new data regarding the phylogeny and classification of *Nannostomus* pencilfish and the order Characiformes, thus providing a theoretical basis for the economic development of aquarium fish markets.

## 2. Materials and Methods

### 2.1. Sample Collection and DNA Extraction

All fish specimens were procured from an aquarium market located in Tianjin, China. Samples of these four fish were identified through morphological and molecular identification, utilizing the resources provided by the WorldFish Center’s FishBase database (https://www.worldfishcenter.org/fishbase, accessed on 11 October 2023) [21] and NCBI (https://www.ncbi.nlm.nih.gov/, accessed on 11 October 2023) [3,8]. The sample used for morphological identification was fresh. In addition, the fish purchased was a normal shape with a complete body. Total DNA was extracted from each fin using the FastPure Cell/Tissue DNA Isolation Mini Kit (Vazyme™, Nanjing, China) and stored in a refrigerator at −20 °C for follow-up.

### 2.2. Mitogenome Sequencing and Assembly

Library construction and sequencing were carried out by Shanghai Personal Biotechnology Co., Ltd. (Shanghai, China) on the NovaSeq X Plus platform (Illumina, CA, USA) following the manufacture’s protocol for 150 bp paired-end reads. The depth of the sequencing was 3×. To generate clean data, low-quality sequences were removed. Clean reads were utilized in the assembly of the complete mitogenomes, using Geneious Prime 2023 using *Lebiasina multimaculata* (AP006766.1) as a template, and both ends of the final assembly were manually examined for any potential overlap in order to construct the circular mitogenomes. The medium sensitivity/speed option was used for the assembly. Consensus sequences were generated with a 50% base call threshold, obtaining the complete mitogenomes.

### 2.3. Sequence Analysis

Conservative domains of the mitogenomes were identified using two tools: BLAST CD-Search (https://www.ncbi.nlm.nih.gov/Structure/cdd/wrpsb.cgi, accessed on 26 November 2023.) and MITOS server (http://mitos.bioinf.uni-leipzig.de/index.py, accessed on 26 November 2023.). Gene maps of the mitogenomes were generated utilizing the CG View server (http://cgview.ca/, accessed on 28 November 2023). The formulas “AT-skew = (A − T)/(A + T)” and “GC-skew = (G − C)/(G + C)” were used to measure nucleotide bias [22]. A heatmap was plotted using heatmap tools in the genescloud platform (https://www.genescloud.cn, accessed on 30 November 2023). The tool was developed from the pheatmap package (V1.0.8), which was slightly modified to improve the layout style [23]. The data were normalized using z-scores. The analysis of relative synonymous codon usage (RSCU), as well as non-synonymous (Ka) and synonymous substitutions (Ks), was conducted using MEGA X software [24]. For RSCU analysis, coding regions were concatenated. tRNA genes were identified using the tRNAscan-SE Search Server (http://lowelab.ucsc.edu/tRNAscan-SE/, accessed on 30 November 2023 [25].

### 2.4. Phylogenetic Analysis

We constructed a concatenated dataset, consisting of the base sequences of the 13 PCGs from a total of 49 species. This dataset was utilized to investigate the phylogenetic relationships within the order Characiformes. Details of the species included in the analysis can be found in Table 1. *Cyprinus carpio* was employed as an outgroup in this study. All operations were performed in PhyloSuite software package v1.2.3 [26]. The alignment of the datasets was performed in batches using MAFFT v7.505 software [27]. MACSE was used to optimize alignments using the classic “Needleman–Wunsch” algorithm [28]. ModelFinder was used to partition the codons and determine the best-fit model for the phylogenetic analyses [29]. Unlike the Akaike Information Criterion (AIC), the Bayesian Information Criterion (BIC) considers the number of samples. When the number of samples is too large, the BIC can effectively prevent the excessive model complexity caused by excessive model precision [30]. The results of the best-fit model are as follows:

Best-fit model of BI according to BIC:

GTR + F + I + G4: *ATP6*, GTR + F + I + G4: *ATP8* + *COX2* + *ND4L*, GTR + F + I + G4: *COX1*, GTR + F + I + G4: *COX3 + ND1*, GTR + F + I + G4: *Cytb*, GTR + F + I + G4: *ND2*, GTR + F + I + G4: *ND3 + ND4 + ND5*, GTR + F + I + G4: *ND6*.

Best-fit model of ML according to BIC:

TIM2 + F + I + G4: *ATP6*, TIM2 + F + I + I + R4: *ATP8* + *COX2* + *ND4L*, TIM2 + F + I + I + R4: *COX1*, TIM2 + F + I + I + R4: *COX3* + *ND1*, TIM2 + F + R5: *Cytb*, TIM2 + F + I + I + R4: *ND2*, GTR + F + I + I + R4: *ND3* + *ND4* + *ND5*, TPM2u + F + R4: *ND6*.

Phylogenetic trees were constructed using Bayesian inference (BI) and maximum likelihood (ML) methods [31,32]. The BI tree was reconstructed using MrBayes 3.2.6 with four Markov chains (three hot chains and one cold chain). Markov chains were run for 1,000,000 generations and were sampled every 100 generations. The consensus trees based on majority rule were assessed by combining the outcomes of duplicated analyses while discarding the first 25% of generations. The ML tree was reconstructed using IQ-TREE with 1000 bootstrap replicates. Phylogenetic trees were visualized and edited using iTOL (https://itol.embl.de/, accessed on 30 November 2023) [33].

## 3. Results

### 3.1. Genome Organization and Composition

**Figure 1:**
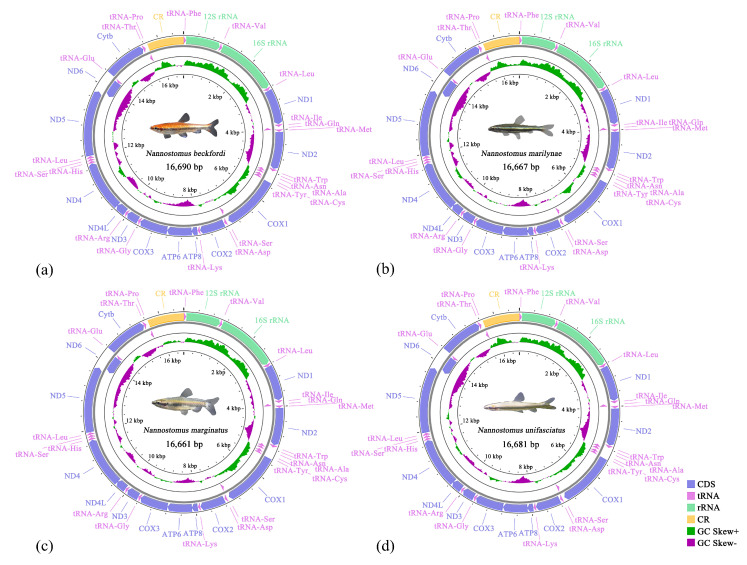
Mitogenomes of *Nannostomus beckfordi* (**a**), *Nannostomus marilynae* (**b**), *Nannostomus marginatus* (**c**), and *Nannostomus unifasciatus* (**d**). Yellow blocks: CR, green blocks: rRNAs, light purple blocks: tRNAs, dark purple blocks: PCGs.

The four complete mitogenomes were classically circular, double-stranded molecules, with sizes of 16,690 bp, 16,667 bp, 16,661 bp, and 16,681 bp (Figure 1). Among these species, *N. marginatus* had the smallest mitogenome, while *N. beckfordi* had the largest. The mitogenomes of the four fish contained 13 PCGs, 22 tRNAs, 2 rRNAs, and 1 noncoding CR. Nine genes, including eight tRNAs and ND6, were encoded on the minor strand, while the remaining genes were located on the major strand (Table 2).

**Figure 2:**
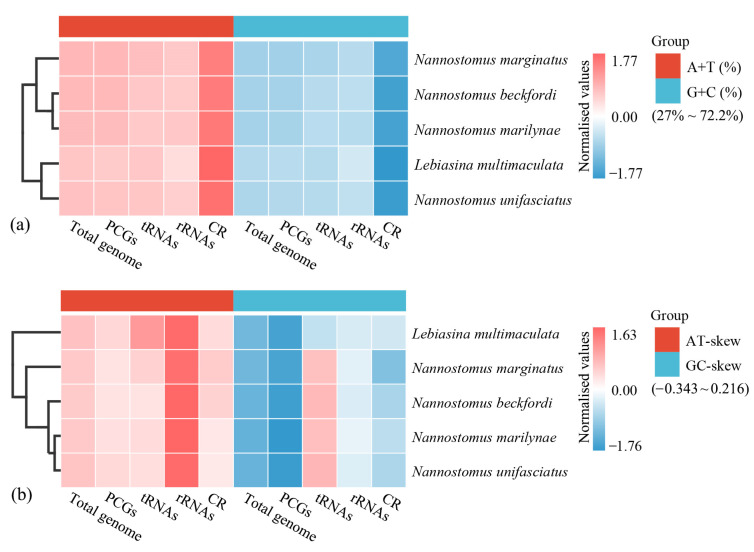
Nucleotide composition of various datasets of mitogenomes. Hierarchical clustering of Lebiasinidae species (y-axis) based on the content (**a**) and skewness (**b**).

**Figure 3:**
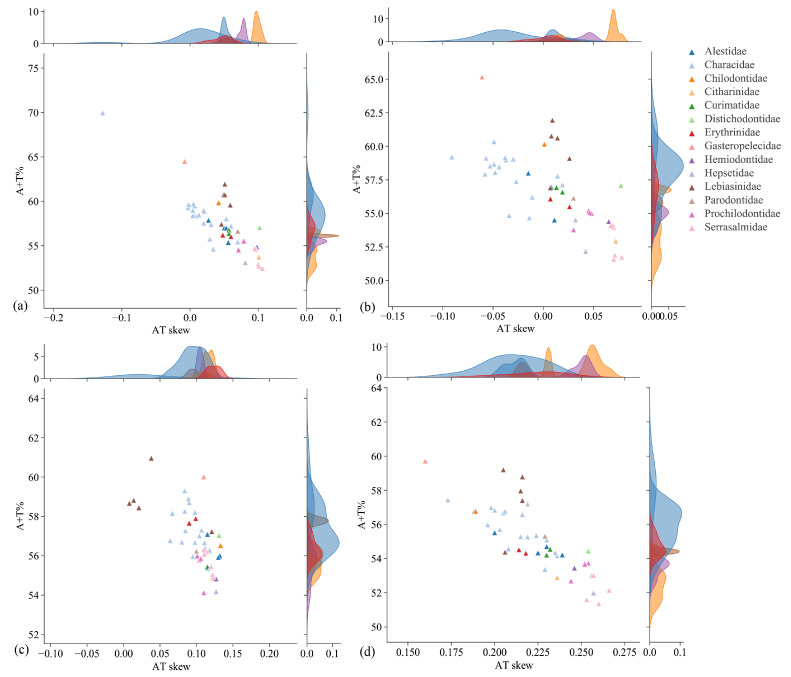
A+T content vs. AT-skew in the 48 mitogenomes of the order Characiformes. (**a**) Total genome; (**b**) PCGs; (**c**) tRNAs; (**d**) rRNAs.

The nucleotide composition analysis suggested that four mitogenomes were biased toward A and T (Figure 2a). In addition, this AT bias (A+T > G+C) was also evident in PCGs, RNAs, and CRs. CRs exhibited the highest A+T content, while PCGs, tRNAs, and rRNAs displayed an A+T content similar to that of the total mitogenomes (Figure 2a). The results of the skewness analysis indicated that the AT skews of four mitogenomes were all positive, while the GC skews were predominantly negative (Figure 2b). Differing from *L. multimaculata* in the same family, the GC skews of tRNAs in *Nannostomus* were all positive. To determine the nucleotide composition of the order Characiformes, the A+T content and AT skew of 48 mitogenomes (including 14 families: Alestidae, Characidae, Chilodontidae, Citharinidae, Curimatidae, Distichodontidae, Erythrinidae, Gasteropelecidae, Hemiodontidae, Hepsetidae, Lebiasinidae, Parodontidae, Prochilodontidae, and Serrasalmidae) were calculated (Table 1 and Figure 3). The 48 Characiformes mitogenomes had a comparable nucleotide composition; the A+T content was always higher than the G+C content in the total genome (52.45~69.97%), PCGs (51.58~65.16%), tRNAs (54.13~60.96%), and rRNAs (51.37~59.71%). The AT skews were almost positive, indicating a higher occurrence of A than T.

Multiple overlaps between adjacent genes were detected (Table 2). Eight gene overlaps were observed in *N. beckfordi* and *N. unifasciatus*, nine in *N. marginatus*, and ten in *N. marilynae*, all ranging from 1 to 10 bp. The largest overlaps among the four mitogenomes were all located between *ATP8* and *ATP6*.

### 3.2. Protein-Coding Genes and Codon Usage

The total lengths of the PCGs in *Nannostomus* were 11,431 bp, 11,433 bp, 11,432 bp, and 11,431 bp, accounting for 68.49% (*N. beckfordi*) to 68.62% (*N. marginatus*) of their total mitogenomes, respectively. All PCGs were encoded on the major strand, except for *ND6* on the minor strand (Figure 1 and Table 2). Among the 13 PCGs presented in these four mitogenomes, *ATP8* exhibited the smallest size at 168 bp, while *ND5* displayed the largest size at 1839 bp.

**Figure 4:**
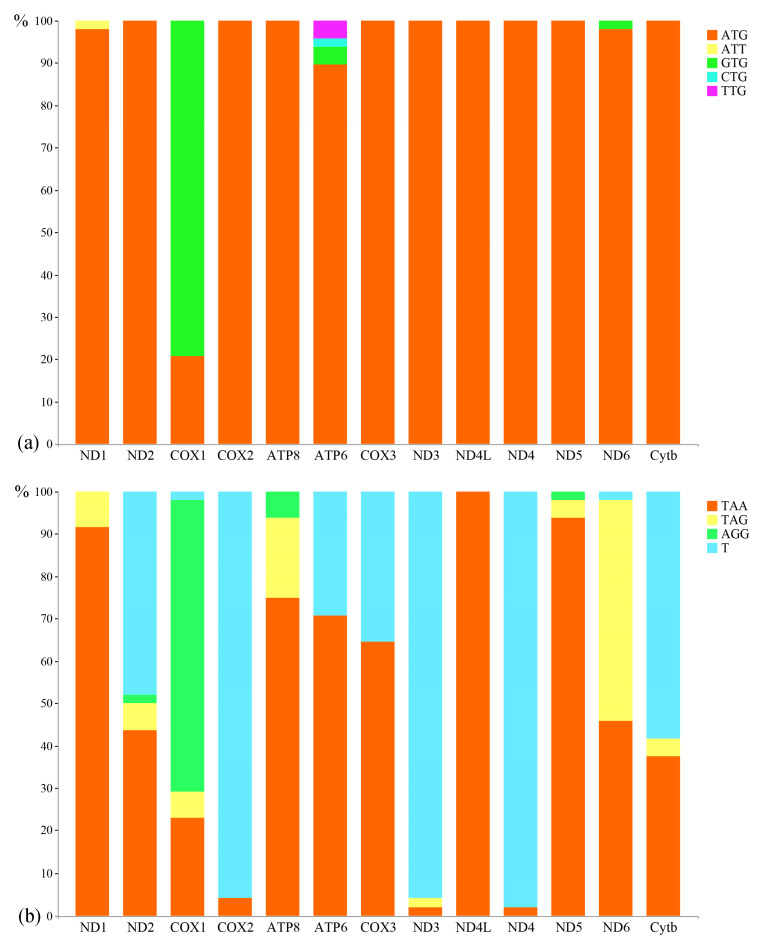
Initiation codon (**a**) and termination codon (**b**) usage for the mitochondrial genome protein-coding genes of 48 Characiformes species.

The majority of PCGs in the four mitogenomes start with the ATG codon, with the exception of *ND1* in *N. marginatus*, which starts with the ATT codon, and *COX1* in all four mitochondrial genomes, which starts with the GTG codon. The termination codon varied across these PCGs, namely TAA, TAG, AGG, and T. Across all mitogenomes, the frequency of the termination codon TAA was consistently higher than that of the other three termination codons, whereas the occurrence of the termination codon AGG was the lowest. The usage of the initiation codon and termination codon in 48 mitogenomes was calculated (Table 1 and Figure 4). The Characiformes species are relatively conservative in their use of initiation codons, and their preferences were almost consistent with those of the four newly sequenced species, starting with ATG (Figure 4). However, COX1 of the Characiformes species mainly started with GTG. All Characiformes species share the termination codons TAA, TAG, AGG, and T (Figure 4). Specifically, *ND1*, *ATP8*, *ATP6*, *COX3*, *ND4L*, and *ND5* predominantly employ TAA as the termination codon, while *COX1* primarily utilizes AGG as the termination codon. Additionally, ND6 mainly uses TAG as the termination codon, and *ND2*, *COX2*, *ND3*, *ND4*, and *Cytb* predominantly use T as the termination codon.

**Figure 5:**
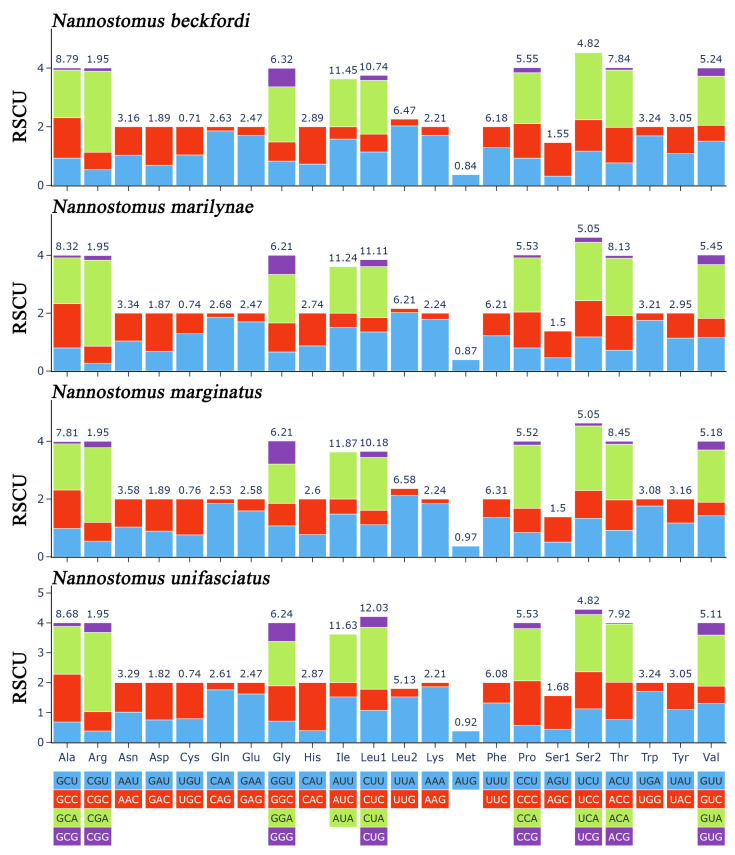
RSCUs of three species of *Nannostomus*; the termination codon is not included.

**Figure 6:**
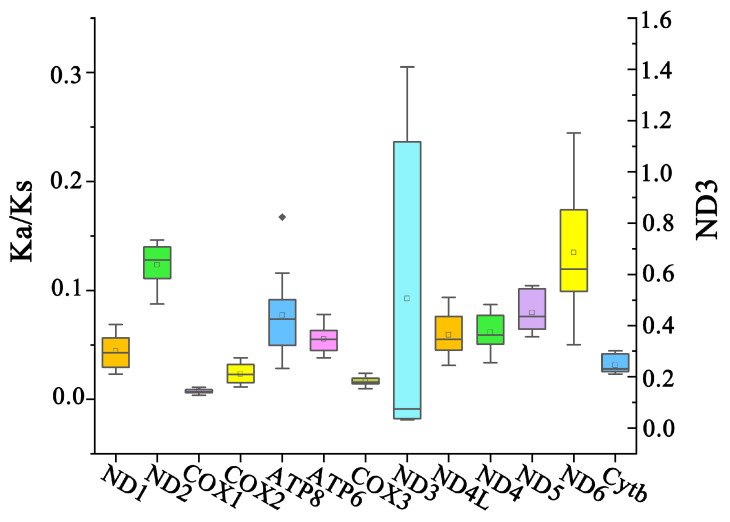
Ka/Ks values for the 13 PCGs of four *Nannostomus* mitogenomes.

An RSCU analysis was conducted to investigate the codon usage patterns in the four mitogenomes of *Nannostomus* (Figure 5). The RSCUs of the four mitogenomes exhibited a high degree of similarity. In addition, RSCU analysis revealed a preference for A/T nucleotides at the third codon position, which was consistent with the biased usage of A+T nucleotides evident in the frequencies of codons. The evolutionary pattern of PCGs in *Nannostomus* was analyzed using Ka/Ks ratios (Figure 6). Apart from that of *ND3*, the Ka/Ks ratios of the PCGs were lower than 1.

### 3.3. rRNA, tRNA Genes, and CR

Two rRNAs, *12S rRNA* and *16S rRNA,* were transcribed from the major strand in the four mitogenomes (Table 2). *12S rRNA* was located between *tRNA-Phe* and *tRNA-Val*, while *16S rRNA* was found between *tRNA-Val* and *tRNA-Leu*. The sizes of the *12S rRNA* ranged from 953 bp to 956 bp, while the *16S rRNA* varied from 1682 bp to 1696 bp in the mitogenomes.

Twenty-two tRNAs (66–76 bp in size) were interspersed in the four mitogenomes altogether, with fourteen from the major strand and eight transcribed from the minor strand (Table 2). The total lengths of the tRNAs were 1563 bp in *N. beckfordi*, 1559 bp in *N. marilynae*, 1565 bp in *N. marginatus*, and 1559 bp in *N. unifasciatus*, accounting for 9.36%, 9.35%, 9.39%, and 9.35% of their total mitogenomes, respectively. 

**Figure 7:**
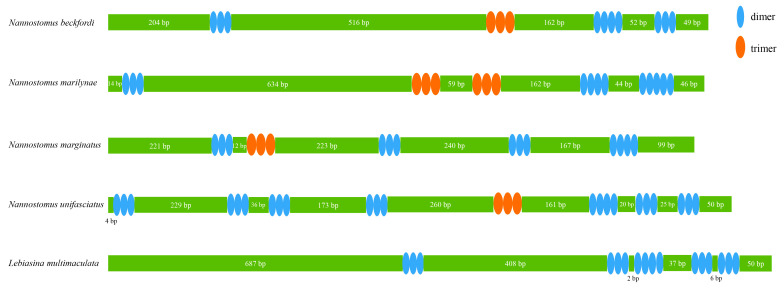
The organization of the control region in five Lebiasinidae mitochondrial genomes. The colored ovals indicate the tandem repeats; the remaining regions are shown with green boxes.

CR was found between the genes *tRNA-Pro* and *tRNA-Phe* in these four mitogenomes. The sizes of CRs in four mitogenomes ranged from 997 bp (*N. marginatus*) to 1012 bp (*N. beckfordi*), accounting for 5.98% to 6.06% of the A+T contents in the CRs of the four mitogenomes, exhibiting consistently higher values than PCGs and RNAs, ranging from 69.88% to 72.20% (Figure 2). Lebiasinidae species had CRs of a similar size, but the length of the repeat units and the number of repeats in them were different (Figure 7). The repeat units of CRs were predominantly dimers and, to a lesser extent, trimers.

### 3.4. Phylogenetic Relationships

**Figure 8:**
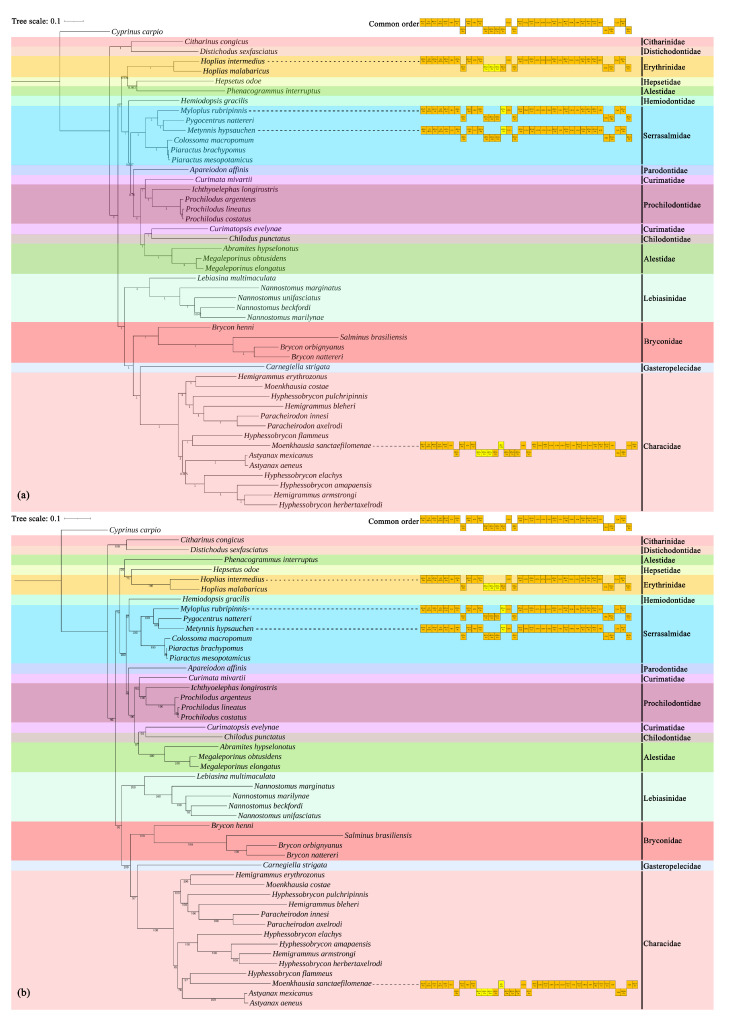
The BI (**a**) and ML (**b**) phylogenetic trees based on the nucleotide datasets for 13 PCGs from the mitogenomes of 49 species, with the common gene order and rearrangement within Characiformes (yellow boxes indicate the events of gene rearrangement).

A total of 48 species from 15 families of the order Characiformes were included in the phylogenetic analyses. Additionally, one species from the order Cypriniformes (*C. carpio*) was selected as the outgroup to establish the phylogenetic trees, our aim being to understand the phylogenetic relationships within the order Characiformes (Table 1). The BI and ML trees shared a similar topological structure, with well-supported values for each clade (Figure 8). Four *Nannostomus* species in this study were clustered together with *L. multimaculata* of the same family. Within the order Characiformes, the families Citharinidae and Distichodontidae diverged with species in other families early in the evolutionary history of Characiformes fishes.

## 4. Discussion

The mitogenomes of the four fish contained 13 PCGs, 22 tRNAs, 2 rRNAs, and 1 noncoding CR, which is typical of vertebrates [10,34,35]. The gene orders of the four fish were found to be identical to the common order of Characiformes, which was previously sequenced [13,36,37]. For nucleotide composition, four mitogenomes had an AT bias (A+T > G+C), which is consistent with previous studies [13,38]. The AT skews were almost positive, indicating a higher occurrence of A than T, as has also been observed in other published Teleostei genomes [10,39]. Some PCGs in the four mitogenomes start with unusual codons, such as ATT and GTG. Previous studies have documented the occurrence of atypical initiation codons in Characiformes, such as *Astyanax paranae* and *Hemigrammus armstrongi* [13,40]. Most of the gene overlap regions appeared between PCGs and PCGs, with the largest overlaps all located between *ATP8* and *ATP6*, consistent with other fish mitogenomes [41,42,43,44]. Apart from *ND3*, the Ka/Ks ratios of other PCGs were lower than 1. This suggests that purifying selection might play a predominant role in shaping the evolutionary patterns of PCGs, meaning that, in most cases, selection eliminates the deleterious mutation, and the protein is unchanged [45]. *COX1* had the lowest average Ka/Ks value, suggesting that it was under drastic selection pressure and evolved slowly [46].

The mitogenome structure of the order Characiformes is generally conserved [47], with infrequent occurrences of gene rearrangement events. Through an examination of the available mitogenomes of the Characiformes species in GenBank (https://www.ncbi.nlm.nih.gov/, accessed on 30 November 2023; Table 1), our investigation revealed instances of gene rearrangement in four species: *Hoplias intermedius*, *Metynnis hypsauchen*, *Moenkhausia sanctaefilomenae*, and *Myloplus rubripinnis*. However, the structure of CRs varied widely among Lebiasinidae species (Figure 7). Previous research has shown that the CRs of fish vary significantly between different species and even within the same species [47,48].

The phylogenetic trees also emphasized the unstable relationships within the family Characidae, which was consistent with previous studies [13,49]. Some Characidae species were clustered with species from other genera (Figure 8). In studies on the genus *Brycon*, some suggest that the genus *Brycon* should be classified under the family Characidae [50,51], while others suggest it should be classified under the family Bryconidae [52,53]. Studies in recent years have mainly supported the classification of the genus *Brycon* belonging to the family Bryconidae [54]. In the phylogenetic analyses in this study, the phylogenetic relationship of the genus *Brycon* with other species in the family Characidae was, indeed, distant. Therefore, our study also supports the inference that the genus *Brycon* should be classified under the family Bryconidae. It is evident that species that have undergone gene rearrangement were clustered together with species of the same family, although *M. sanctaefilomenae* did not cluster with other species in the same genus. Previous studies have indicated that phylogenetic trees based on PCGs are more stable and representative than those based on RNAs [13,55,56]. Therefore, the phylogenetic analyses based on PCGs in this study suggest that gene rearrangement would not significantly impact the phylogenetic relationships within the order Characiformes. Although the Characiformes mitogenome is relatively conserved [47], gene rearrangement events have been discovered in many taxa. In addition, there are still a large number of Characiformes species whose complete mitogenomes have not yet been published, and our knowledge on the structure of Characiformes mitogenomes, especially the pattern and underlying mechanisms of gene rearrangements, is far from comprehensive. Therefore, it is necessary to obtain mitogenome data on more species of the order Characiformes. The selection of species from the genus *Nannostomus* in this study, as well as other species from the Lebiasinidae family in previous studies, was limited, thereby hindering the ability to conduct a comprehensive analysis. Consequently, to enhance our comprehension of the relationships within this family, it would be helpful to incorporate a broader range of species in forthcoming research endeavors.

## 5. Conclusions

In summary, the four mitogenomes exhibited a typical circular structure, with the overall sizes varying from 16,661 bp to 16,690 bp, containing 13 PCGs, 2 rRNAs, 22 tRNAs, and 1 CR. Nucleotide composition analysis suggested that the mitochondrial sequences were biased towards A and T. The gene order of the four *Nannostomus* pencilfish was similar to that of other Osteichthyes fish. Phylogenetic analyses support the current classification of the family Lebiasinidae. The phylogenetic analyses in this study suggest that gene rearrangement would not significantly impact the phylogenetic relationships within the order Characiformes. These findings provide new data on the phylogeny and classification of the order Characiformes, thereby establishing a theoretical foundation for the sustainable development of aquarium fish markets.

## Figures and Tables

**Table 1:** The mitogenomes of Characiformes and Cypriniformes used in this study.

Order.	Family	Genus	Species	Size (bp)	Accession No.
Characiformes	Alestidae	*Abramites*	*Abramites hypselonotus*	16,685	MW541938.1
		*Megaleporinus*	*Megaleporinus elongatus*	16,774	KU980144.1
			*Megaleporinus obtusidens*	16,682	KY825191.1
		*Phenacogrammus*	*Phenacogrammus interruptus*	16,652	AB054129.1
	Bryconidae	*Brycon*	*Brycon henni*	16,885	KP027535.1
			*Brycon nattereri*	16,837	MT428073.1
			*Brycon orbignyanus*	16,802	KY825192.1
		*Salminus*	*Salminus brasiliensis*	17,721	KM245047.1
	Characidae	*Astyanax*	*Astyanax aeneus*	16,769	BK013055.1
			*Astyanax mexicanus*	16,768	BK013062.1
		*Hemigrammus*	*Hemigrammus armstrongi*	16,789	MW742324.1
			*Hemigrammus bleheri*	17,021	LC074360.1
			*Hemigrammus erythrozonus*	16,710	MT484070.1
		*Hyphessobrycon*	*Hyphessobrycon amapaensis*	17,824	MW742322.1
			*Hyphessobrycon elachys*	17,224	MW315747.1
			*Hyphessobrycon flammeus*	16,008	MW315748.1
			*Hyphessobrycon herbertaxelrodi*	17,417	MT769327.1
			*Hyphessobrycon pulchripinnis*	17,618	MW331227.1
		*Moenkhausia*	*Moenkhausia costae*	15,811	MW366831.1
			*Moenkhausia sanctaefilomenae*	18,437	MW407181.1
		*Paracheirodon*	*Paracheirodon axelrodi*	17,100	AB898197.1
			*Paracheirodon innesi*	16,962	KT783482.1
	Chilodontidae	*Chilodus*	*Chilodus punctatus*	16,869	AP011984.1
	Citharinidae	*Citharinus*	*Citharinus congicus*	16,453	AP011985.1
	Curimatidae	*Curimata*	*Curimata mivartii*	16,705	KP025764.1
		*Curimatopsis*	*Curimatopsis evelynae*	16,779	AP011988.1
	Distichodontidae	*Distichodus*	*Distichodus sexfasciatus*	16,555	AB070242.1
	Erythrinidae	*Hoplias*	*Hoplias intermedius*	16,629	KU523584.1
			*Hoplias malabaricus*	16,638	AP011992.1
	Gasteropelecidae	*Carnegiella*	*Carnegiella strigata*	17,852	AP011983.1
	Hemiodontidae	*Hemiodopsis*	*Hemiodopsis gracilis*	16,731	AP011990.1
	Hepsetidae	*Hepsetus*	*Hepsetus odoe*	16,803	AP011991.1
	Lebiasinidae	*Lebiasina*	*Lebiasina multimaculata*	16,899	AP006766.1
		*Nannostomus*	*Nannostomus beckfordi*	16,690	OR857846
			*Nannostomus marilynae*	16,667	OR857847
			*Nannostomus marginatus*	16,661	OR857848
			*Nannostomus unifasciatus*	16,681	OR857849
	Parodontidae	*Apareiodon*	*Apareiodon affinis*	16,679	AP011998.1
	Prochilodontidae	*Ichthyoelephas*	*Ichthyoelephas longirostris*	16,840	KP025763.1
		*Prochilodus*	*Prochilodus argenteus*	16,697	KR014816.1
			*Prochilodus costatus*	16,699	KR014817.1
			*Prochilodus lineatus*	16,699	KM245045.1
	Serrasalmidae	*Colossoma*	*Colossoma macropomum*	16,703	KP188830.1
		*Metynnis*	*Metynnis hypsauchen*	16,737	MH358334.1
		*Myloplus*	*Myloplus rubripinnis*	16,662	MH358336.1
		*Piaractus*	*Piaractus brachypomus*	16,722	KJ993871.2
			*Piaractus mesopotamicus*	16,722	KM245046.1
		*Pygocentrus*	*Pygocentrus nattereri*	16,706	AP012000.1
Cypriniformes	Cyprinidae	*Cyprinus*	*Cyprinus carpio*	16,592	OL693871.1

**Table 2:** General features of the mitogenomes of *Nannostomus beckfordi*, *Nannostomus marilynae*, *Nannostomus marginatus*, and *Nannostomus unifasciatus*.

Gene	Position		Size (bp)	Orientation	Codon		Intergenic Nucleotides (bp)
	From	To			Initiation	Termination	
*tRNA-Phe*	1/1/1/1	70/70/71/70	70/70/7/70	+/+/+/+			0/0/0/0
*12S rRNA*	71/71/72/71	1026/1025/1024/1024	956/955/953/954	+/+/+/+			0/0/0/0
*tRNA-Val*	1026/1025/1024/1024	1097/1096/1095/1095	72/72/72/72	+/+/+/+			−1/−1/−1/−1
*16S rRNA*	1098/1097/1096/1096	2793/2786/2777/2785	1696/1690/1682/1690	+/+/+/+			0/0/0/0
*tRNA-Leu*	2796/2789/2779/2788	2871/2864/2854/2863	76/76/76/76	+/+/+/+			2/2/1/2
*ND1*	2872/2865/2852/2864	3846/3839/3826/3838	975/975/975/975	+/+/+/+	ATG/ATG/ATT/ATG	TAG/TAA/TAA/TAA	0/0/−3/0
*tRNA-Ile*	3851/3843/3832/3842	3922/3914/3903/3913	72/72/72/72	+/+/+/+			4/3/5/3
*tRNA-Gln*	3921/3913/3902/3912	3991/3983/3972/3982	71/71/71/71	−/−/−/−			−2/−2/−2/−2
*tRNA-Met*	3992/3983/3973/3986	4061/4052/4042/4055	70/70/70/70	+/+/+/+			0/−1/0/3
*ND2*	4062/4053/4043/4056	5106/5099/5087/5100	1045/1047/1045/1045	+/+/+/+	ATG/ATG/ATG/ATG	T/TAG/T/T	0/0/0/0
*tRNA-Trp*	5107/5098/5088/5101	5178/5169/5160/5172	72/72/73/72	+/+/+/+			0/−2/0/0
*tRNA-Ala*	5182/5173/5163/5176	5250/5241/5231/5244	69/69/69/69	−/−/−/−			3/3/2/3
*tRNA-Asn*	5252/5243/5233/5246	5324/5315/5305/5318	73/73/73/73	−/−/−/−			1/1/1/1
*tRNA-Cys*	5354/5346/5337/5349	5420/5411/5402/5414	67/66/66/66	−/−/−/−			29/30/31/30
*tRNA-Tyr*	5421/5412/5403/5415	5490/5481/5470/5483	70/70/68/69	−/−/−/−			0/0/0/0
*COX1*	5492/5483/5472/5485	7048/7039/7028/7041	1557/1557/1557/1557	+/+/+/+	GTG/GTG/GTG/GTG	AGG/AGG/AGG/AGG	1/1/1/1
*tRNA-Ser*	7040/7031/7020/7033	7110/7101/7090/7103	71/71/71/71	−/−/−/−			−9/−9/−9/−9
*tRNA-Asp*	7115/7106/7096/7108	7184/7175/7165/7177	70/70/70/70	+/+/+/+			4/4/5/4
*COX2*	7198/7189/7180/7192	7888/7879/7870/7882	691/691/691/691	+/+/+/+	ATG/ATG/ATG/ATG	T/T/T/T	13/13/14/14
*tRNA-Lys*	7889/7880/7871/7883	7962/7953/7944/7956	74/74/74/74	+/+/+/+			0/0/0/0
*ATP8*	7964/7955/7946/7958	8131/8122/8113/8125	168/168/168/168	+/+/+/+	ATG/ATG/ATG/ATG	TAA/TAA/TAA/TAA	1/1/1/1
*ATP6*	8122/8113/8104/8116	8805/8796/8787/8799	684/684/684/684	+/+/+/+	ATG/ATG/ATG/ATG	TAA/TAA/TAA/TAA	−10/−10/−10/−10
*COX3*	8805/8796/8787/8799	9590/9581/9572/9584	786/786/786/786	+/+/+/+	ATG/ATG/ATG/ATG	TAA/TAA/TAA/TAA	−1/−1/−1/−1
*tRNA-Gly*	9590/9581/9572/9584	9662/9652/9647/9655	73/72/76/72	+/+/+/+			−1/−1/−1/−1
*ND3*	9663/9653/9648/9656	10,008/9998/9993/10,001	346/346/346/346	+/+/+/+	ATG/ATG/ATG/ATG	T/T/T/T	0/0/0/0
*tRNA-Arg*	10,009/9999/9994/10,002	10,079/10,067/10,063/10,071	71/69/70/70	+/+/+/+			0/0/0/0
*ND4L*	10,080/10,068/10,064/10,072	10,376/10,364/10,360/10,368	297/297/297/297	+/+/+/+	ATG/ATG/ATG/ATG	TAA/TAA/TAA/TAA	0/0/0/0
*ND4*	10,370/10,358/10,354/10,362	11,750/11,738/11,734/11,742	1381/1381/1381/1381	+/+/+/+	ATG/ATG/ATG/ATG	T/T/T/T	−7/−7/−7/−7
*tRNA-His*	11,752/11,740/11,735/11,744	11,821/11,808/11,804/11,813	70/69/70/70	+/+/+/+			1/1/0/1
*tRNA-Ser*	11,822/11,809/11,805/11,814	11,889/11,876/11,872/11,881	68/68/68/68	+/+/+/+			0/0/0/0
*tRNA-Leu*	11,891/11,878/11,874/11,883	11,963/11,950/11,946/11,955	73/73/73/73	+/+/+/+			1/1/1/1
*ND5*	11,964/11,951/11,947/11,956	13,802/13,789/13,785/13,794	1839/1839/1839/1839	+/+/+/+	ATG/ATG/ATG/ATG	TAA/TAA/TAA/TAA	0/0/0/0
*ND6*	13,799/13,786/13,782/13,791	14,317/14,304/14,300/14,309	519/519/519/519	−/−/−/−	ATG/ATG/ATG/ATG	TAG/TAA/TAG/TAA	−4/−4/−4/−4
*tRNA-Glu*	14,318/14,305/14,301/14,310	14,386/14,372/14,369/14,377	69/68/69/68	−/−/−/−			0/0/0/0
*Cytb*	14,392/14,378/14,375/14,383	15,534/15,520/15,518/15,525	1143/1143/1144/1143	+/+/+/+	ATG/ATG/ATG/ATG	TAA/TAA/T/TAA	5/5/5/5
*tRNA-Thr*	15,536/15,522/15,519/15,527	15,607/15,595/15,592/15,599	72/74/74/73	+/+/+/+			1/1/0/1
*tRNA-Pro*	15,609/15,597/15,596/15,601	15,678/15,666/15,664/15,670	70/70/69/70	−/−/−/−			1/1/3/1
CR	15,679/15,667/15,665/15,671	16,690/16,667/16,661/16,681	1012/1001/997/1011				0/0/0/0

## Data Availability

DNA sequences: GenBank accession number OR857846 for *Nannostomus beckfordi*, OR857847 for *Nannostomus marilynae*, OR857848 for *Nannostomus marginatus*, and OR857849 for *Nannostomus unifasciatus*.
